# Long-term intake of saccharin decreases post-absortive energy expenditure at rest and is associated to greater weight gain relative to sucrose in wistar rats

**DOI:** 10.1186/s12986-017-0165-7

**Published:** 2017-02-20

**Authors:** Denise Entrudo Pinto, Kelly Carraro Foletto, Ramiro Barcos Nunes, Pedro Dal Lago, Marcello Casaccia Bertoluci

**Affiliations:** 1 Programa de Pós-Graduação em Medicina: Ciências Médicas, Universidade Federal do Rio Grande do Sul-UFRGS, Rua Ramiro Barcelos, n° 2400, CEP 90035-003, Bairro Rio Branco, Porto Alegre, RS Brazil; 2 Laboratório de Fisiologia Experimental, Universidade Federal de Ciências da Saúde de Porto Alegre-UFCSPA, Rua Sarmento Leite, n° 245, CEP 90050-170, Bairro Centro, Porto Alegre, RS Brazil; 3 Serviço de Medicina Interna, Hospital de Clínicas de Porto Alegre-Universidade Federal do Rio Grande do Sul, Rua Ramiro Barcelos, n° 2350, CEP 90035-903, Bairro Rio Branco, Porto Alegre, RS Brazil

**Keywords:** Non-nutritive sweeteners, Saccharin, Sucrose, Weight gain, Energy expenditure, Oxygen consumption

## Abstract

**Background:**

Non-nutritive sweeteners (NNS) have been associated with increased prevalence of obesity. In previous studies, we demonstrated that saccharin could induce an increase in weight gain either when compared to sucrose or to a non-sweetened control at a similar total caloric intake. These data raised the hypothesis that reduced energy expenditure (EE) could be a potential mechanism explaining greater weight gain with saccharin use in rats. The aim of the present study was to compare long-term energy expenditure at rest between rats using saccharin or sucrose and correlate it with weight gain. ﻿

**Methods:**

In the present study, we examine the potential impact of saccharin compared to sucrose in the EE of Wistar rats. In a controlled experiment of 17 weeks, 24 Wistar rats were divided into 2 groups: saccharin-sweetened yogurt (SAC) or sucrose-sweetened yogurt (SUC), plus a free chow diet. Only rats that consumed at least 70% of the offered yogurt were included. EE (kcal/day) was determined at rest through open circuit indirect calorimetry system in the early post-absorptive period with determinations of both VO_2_ consumption and CO_2_ production. Measurements were evaluated at baseline, 5 and 12 weeks of dietary intervention. Weight gain, caloric intake (from yogurt, from chow and total) were determined weekly.

**Results:**

Body weight and EE were similar between groups at baseline: (*p* = .35) and (*p* = .67) respectively. At the end of the study, SAC increased total weight gain significantly more in relation to SUC (*p* = .03). Cumulative total caloric intake (yogurt plus chow) was similar between groups during the whole period (*p* = .54). At 12 weeks, the EE was smaller in SAC compared to SUC (*p* = .009). Considering both groups, there was a strong negative correlation between total weight gain and change in EE observed [*r*(20) = −.61, *p* = .003]. However, when analyzing the groups separately we found that SUC maintained this inverse correlation [*r*(8) = −.68, *p* = .03], while SAC did not [*r*(10) = −.33, *p* = .29].

**Conclusion:**

These data support the hypothesis that long-term use of saccharin may blunt post-absorptive EE at rest in Wistar rats, which is related to weight gain. On the other hand, long-term sucrose intake can increase energy expenditure in rats. This effect combined can explain, at least partially, the weight gain increases associated to saccharin in relation to sucrose in these animals.

## Background

Non-nutritive sweeteners (NNS) have been associated with an increase in prevalence of obesity [1]. Epidemiological studies have shown that the use of NNS containing products correlates with the incidence of adiposity, metabolic syndrome (MS) [2, 3], type 2 diabetes mellitus (DM2) and cardiovascular disease [4, 5]. A clear cause and effect relationship, however, cannot be established.

The use of NNS, including saccharin, has been shown to interfere in the regulation of appetite and weight gain, which was demonstrated in some experimental studies and in randomized clinical trials [6–8]. Swithers and Davidson [9] conducted a series of experiments demonstrating that the use of saccharin resulted in reduced ability to compensate calories, providing a higher total caloric intake, greater weight gain and adiposity, when compared to the use of glucose.

In previous studies, we demonstrated that, in Wistar rats, saccharin can induce greater weight gain both in relation to sucrose [10] and to non-sweetened supplements [11], in spite of a similar total caloric intake. These experiments informed that, when rats receive saccharin-sweetened yogurt along with free chow intake, a compensatory increase in chow intake occurs. Nevertheless, the caloric deficit was perfectly compensated in a way that the total caloric intake (the sum of chow plus supplemented yogurt) was similar between groups. Despite this fact, significant increases in weight gain in the saccharin group could not be explained by increases in total caloric intake.

Mechanisms to explain why saccharin is associated with weight gain in rats are still not clear. One natural inference is to speculate that energy expenditure might be reduced in rats taking saccharin in relation to sucrose. Sprague–Dawley rats that were previously exposed to saccharin presented a smaller rise in the core-temperature after a standard meal when compared to rats pre-exposed to glucose, indicating that saccharin may interfere in heat production after meals and, therefore, in caloric expenditure [9].

In the present study, we hypothesized that the increase in long-term weight gain due to saccharin relatively to sucrose may be related to energy expenditure reduction induced by saccharin. We conducted a 17-week experiment with Wistar rats consuming either saccharin or sucrose along with a free chow diet, and compared the energy expenditure (EE) between groups at rest, in the post-absortive period at baseline, 5 and 12 weeks.

## Methods

### Study design and animals

In a 17-week controlled experiment, 24 adult male Wistar rats weighing ~200 g were randomly divided equally into 2 groups according to the type of sweetener: saccharin-sweetened yogurt (SAC group) or sucrose-sweetened yogurt (SUC group), besides standard chow and water *ad lib.* Total weight gain and caloric intake were measured weekly. EE was measured at baseline, 5 weeks and 12 weeks. The researchers were blinded to the assigned groups.

Animals were individually housed in translucent polypropylene plastic cages, with controlled humidity (65-70%) at room temperature (22 ± 1 °C), maintained through a 12 h-light and dark cycle.

Procedures were conducted in accordance with the Principles of Laboratory Animal Care (NIH publication No. 86–23, revised 1985) and guidelines of the National Research Council Committee for the Care and Use of Laboratory Animals [12] in the same degree as the Brazilian law for the scientific use of animals. Study protocol was approved by the ethics committee for experimental procedures of Universidade Federal de Ciências da Saúde de Porto Alegre (UFCSPA) and by the ethics committee for experimental procedures of Hospital de Clínicas de Porto Alegre (HCPA). This project was carried out in the Laboratory of Experimental Physiology at UFCSPA.

### Dietary manipulation

All rats received standard chow pellets *ad lib* containing 2.93 kcal/g, (Nuvital CR-1, Nuvilab™). Additional chow was added to the top of the cages every 24 h as needed, and chow rest was weighed and registered once a week. For weighing, all chow was removed and weighed with an electronic precision scale (AS 5500, Marte™, SP, Brazil). The intake was calculated and registered weekly. The largest solid pellets were allocated in the grid feeders. A crumb collector was installed on the outside of the cage to minimize losses. Cages were carefully monitored for any evidence of chow spillage and crumbs were considered for the control of chow intake.

Sweetened yogurt supplements were prepared according to our former protocol [10, 11]. In short, it included 20 ml of standardized low-fat yogurt (Nestlé™, SP, Brazil – containing per unit of 160 g 57 kcal, 7.8 grams of carbohydrate, 5.8 grams of protein and 0 grams of total fat) to which it was added either dry sucrose (União™, SP, Brazil) or sodium-saccharin (Zero-Cal™, SP, Brazil) to include 20% sucrose solution and .3% saccharin solutions respectively. Yogurt supplements were offered 22 h daily, from 11 AM to 9 AM, 7 days a week during the whole experiment. Yogurt was offered in special bottles with adapted beaks to avoid leakage. Additionally, 15 ml of pure water was added into yogurt to dilute and adjust viscosity in order to allow easier drinking. Caloric densities of sucrose-sweetened yogurt and saccharin-sweetened yogurt were: .63 kcal/ml (~170 kcal/wk) and .24 kcal/ml (~60 kcal/wk), respectively. Rats that drank less than 70% of sweetened yogurt were excluded from the study. The yogurt bottles were also checked for any sign of leaking or clogging.

### Caloric intake determination

Mean caloric intake was determined by the week difference between the weight of offered chow and the weight of the chow left in the grid, corrected by the rat weight at the end of the week using the following formula:

$$ \mathrm{Mean}\ \mathrm{Caloric}\ \mathrm{Intake}\ \mathrm{of}\ {\mathrm{Yogurt}}_{1\hbox{-} 17\mathrm{wk}}\ \left(\mathrm{kcal}/\mathrm{g}/\mathrm{wk}\right) = \left(\frac{\sum\ \mathrm{k}\mathrm{cal}\ \mathrm{of}\ {\mathrm{yogurt}}_{1\mathrm{wk}}}{{\mathrm{weight}}_{1\mathrm{wk}}} + \cdots + \frac{\sum\ \mathrm{k}\mathrm{cal}\ \mathrm{of}\ {\mathrm{yogurt}}_{17\mathrm{wk}}}{{\mathrm{weight}}_{17\mathrm{wk}}}\right) \div 17\mathrm{w}\mathrm{k} $$

$$ \mathrm{Mean}\ \mathrm{Caloric}\ \mathrm{Intake}\ \mathrm{of}\ {\mathrm{Chow}}_{1\hbox{-} 17\mathrm{wk}}\ \left(\mathrm{kcal}/\mathrm{g}/\mathrm{wk}\right) = \left(\frac{\sum\ \mathrm{k}\mathrm{cal}\ \mathrm{of}\ {\mathrm{chow}}_{1\mathrm{wk}}}{{\mathrm{weight}}_{1\mathrm{wk}}} + \cdots + \frac{\sum\ \mathrm{k}\mathrm{cal}\ \mathrm{of}\ {\mathrm{chow}}_{17\mathrm{wk}}}{{\mathrm{weight}}_{17\mathrm{wk}}}\right) \div 17\mathrm{w}\mathrm{k} $$

$$ \mathrm{Mean}\ \mathrm{Total}\ \mathrm{Caloric}\ {\mathrm{Intake}}_{1\hbox{-} 17\mathrm{wk}}\ \left(\mathrm{kcal}/\mathrm{g}/\mathrm{wk}\right) = \left(\frac{\sum\ \mathrm{k}\mathrm{cal}\mathrm{f}\ {\mathrm{total}}_{1\mathrm{wk}}}{{\mathrm{weight}}_{1\mathrm{wk}}} + \cdots + \frac{\sum\ \mathrm{k}\mathrm{cal}\ {\mathrm{total}}_{17\mathrm{wk}}}{{\mathrm{weight}}_{17\mathrm{wk}}}\right) \div 17\mathrm{w}\mathrm{k} $$

Cumulative total caloric intake (including yogurt and chow), cumulative caloric intake of yogurt and cumulative caloric intake of chow were calculated by the sum of calories ingested along each week, and corrected by the corresponding rat weight at the end of each week. These data were calculated in the cumulative mode for the 17-week period and were expressed as kcal/g of rat.

### Body weight

Rats were weighed weekly at the same time in the morning, using an electronic precision scale, suitable for weighing animals in motion (AS 5500, Marte™, SP, Brazil). The absolute weight gain after 17 weeks of dietary intervention was calculated as follows:

$$ \mathrm{Total}\ \mathrm{weight}\ \mathrm{gain}\left(\mathrm{g}\right) = \mathrm{body}\ {\mathrm{weight}}_{17\mathrm{wk}}\ \hbox{-}\ \mathrm{body}\ {\mathrm{weight}}_{\mathrm{baseline}} $$

Cumulative weight gain was calculated by the subtraction of the basal weight from the weight obtained every week, and expressed in grams.

### Indirect calorimetric

The protocol of indirect calorimetric was adapted from Rodrigues et al. [13]. Measurements were made using an open-system oxygen gas chamber, at controlled room temperature of 21 °C. The metabolic chamber was connected to a vacuum pump that generates 2.5 L/min O_2_ flow. Oxygen flow was determined using O_2_ and CO_2_ analyzer (AVS Projects^TM^, São Carlos, SP, Brazil). Animals were placed into a gas chamber for 15 min to allow stabilization. After that, data started to be recorded for 5 min.

Tests were performed 2-3 h in the post-absorptive period at rest in the baseline, week 5 and week 12. All chow and yogurt were removed 2 h before experiments. All tests were performed in duplicate, being repeated in the immediate next following day. The mean of 2 results was considered for statistical analysis.

### Determination of respiratory variables

#### Oxygen Consumption (VO_2_)

VO_2_ was calculated according to the flow of environment air pumped into the metabolic chamber using the formula:

$$ {\mathrm{VO}}_2\ \left(\mathrm{ml}/{\mathrm{kg}}^{\hbox{-} 1}/{ \min}^{\hbox{-} 1}\right) = \mathrm{P}\mathrm{F}\ \frac{\left(\mathrm{A}\ \hbox{-}\ \mathrm{E}\right)}{\mathrm{BW}} $$

Where:

"PF" is the measured flow through the metabolic chamber (2500 ml/min);

"A" is the fraction of oxygen entering the chamber (environment air);

"E" is the fraction of effluent oxygen;

"BW" is the animal body weight in grams.

### Carbone Dioxide Production (VCO_2_)

The VCO_2_ production determination was obtained from the carbon dioxide removal detected by a sensor at the chamber output expressed in ml/kg/min.

Both VO_2_ and VCO_2_ were calculated according to ACQUAD software.

### Respiratory Exchange Ratio (RER)

The RER, also known as respiratory quotient, was calculated according to the formula:

$$ \mathrm{R}\mathrm{E}\mathrm{R} = \frac{{\mathrm{VCO}}_2}{{\mathrm{VO}}_2} $$

### Energy Expenditure (EE)

EE was estimated using the Weir equation [14] extrapolated to 24-h:

$$ \mathrm{E}\mathrm{E}\ \left(\mathrm{kcal}/\mathrm{day}\right) = \left(3.94{\mathrm{VO}}_2 + 1.11{\mathrm{VCO}}_2\right) \times 1440 $$

Where VO_2_ and VCO_2_ were expressed in L/min and 1440 is the number of minutes in a day.

The change in EE was determined as the difference in EE at the end of 12 week and at baseline, calculated according to the formula:

$$ \mathrm{Change}\ \mathrm{in}\ \mathrm{E}\mathrm{E}\ \left(\mathrm{kcal}/\mathrm{day}\right) = {\mathrm{EE}}_{12\mathrm{wk}}\ \hbox{-}\ {\mathrm{EE}}_{\mathrm{baseline}} $$

### Statistical analysis

The variables: total body weight gain, the area under the curve (AUC)_,_ mean caloric intake of chow, yogurt, and total caloric intake (chow plus sweetened-yogurt) were compared using *t*-tests.

ANOVA of repeated measures was used to compare the effect over time of cumulative weight gain, cumulative caloric intake (of sweetened-yogurt, of chow and total), RER, VO_2_, VCO_2_ and EE.

We used Pearson’s correlation coefficient (r) to evaluate the association between weight gain and change in energy expenditure over the entire experiment and it was presented graphically and numerically with r and *p* values. Reported values are mean and standard error ± SE, and *p* ˂ .05 was taken as significant for all analysis. Statistical analyses were performed using SPSS™ 21 (IBM Coorporation, Armonk, NY, USA). AUCs were obtained through software NCSS™ 2007 (Number Cruncher Statistical Systems, Kaysville, UT, USA), calculated by the trapezoidal method.

## Results

### Total weight gain

**Fig. 1:**
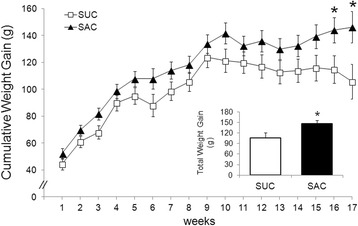
Cumulative weight gain (g) over 17 weeks, determined by ANOVA of repeated measures. There was statistical difference from the 16th weeks [*F*(3.01, 60.26) = 2.99, *p* = .038]. Bar chart indicate analysis by Student's *t*-test [t(20) = − 2.32, *p =* .03]. Group labels: SAC (Saccharin, *n* = 12) and SUC (Sucrose, *n* = 10). Error bars indicate SE and asterisk (*) indicates *p* < .05

At baseline, body weight (g) did not differ between groups: SUC: 219.00 ± 6.74 *vs* SAC: 207.33 ± 9.48); *t*(20) = .96, *p* = .35. After 17 weeks, SAC showed greater weight gain compared to SUC, respectively: 146.25 ± 10.30 *vs* 105.70 ± 14.62; *t*(20) = −2.32, *p* = .03. This corresponded to an increase from baseline of 71% and 48% in SAC and SUC, respectively. When we analyzed the weight gain of each week, we verified that the statistical significance occurred from the 16th week [*F*(3.01, 60.26) = 2.99, *p* = .038] (Fig. 1).

### Caloric intake

#### Sweetened-yogurt intake

Calories derived from sweetened-yogurt in SUC were 2.8 times greater than in SAC due to the higher caloric density in sucrose-sweetened yogurt; *t*(9.87) = 18.10, *p <* .001.

**Fig. 2:**
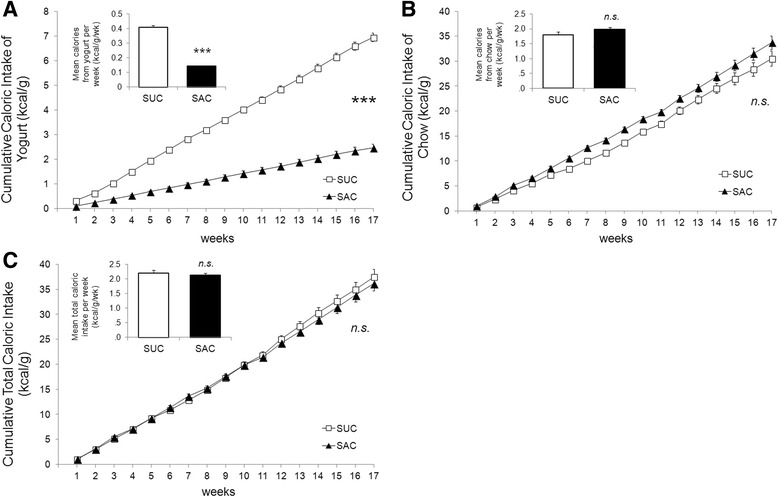
**a** Cumulative caloric intake of sweetened yogurt corrected by rat weight (kcal/g) over 17 weeks, determined by ANOVA of repetead measures [*F*(1.13, 22.61) = 287.64, *p* < .001]. Bar chart indicate analysis by Student's *t*-test [t(18.10) = 9.87, *p* < .001]. Asterisks indicate statistical difference in both analyzes. Group labels: SAC (Saccharin, *n* = 12) and SUC (Sucrose, *n* = 10). Error bars indicate SE and asterisks (***) indicate *p* < .001. **b** Cumulative caloric intake of chow corrected by rat weight (kcal/g) over 17 weeks, determined by ANOVA of repeated measures [*F*(1.42, 28.44) = 1.70, *p* = .21]. Bar chart indicate analysis by Student's *t*-test [t(20) = −1.56, *p* = .13]. Group labels: SAC (Saccharin, *n* = 12) and SUC (Sucrose, *n* = 10). Error bars indicate SE. There was no statistical difference in both analyzes. **c** Cumulative total caloric intake corrected by rat weight (kcal/g) over 17 weeks, determined by ANOVA of repeated measures [*F*(1.38, 27.64) = 1.05, *p* = .34]. Bar chart indicate analysis by Student's *t*-test [t(20) = .63, *p* = .54]. Group labels: SAC (Saccharin, *n* = 12) and SUC (Sucrose, *n* = 10). Error bars indicate SE. There was no statistical difference in both analyzes

The longitudinal and cumulative analysis showed statistically difference from the first week [*F*(1.13, 22.61) = 287.64, *p* < .001] (Fig. 2a). Two rats were excluded from the SUC group due to yogurt intake less than 70%. There were no differences between groups in yogurt acceptance in the remaining rats, (80% ± .87); *t*(20) = −1.54, *p* = .14.

### Chow intake

The mean caloric intake from chow tended to be greater in SAC than in SUC, corresponding to a 10% compensatory increment, although not statistically significant; *t*(20) = −1.56, *p* = .13. In the repeated measure analysis, the time x group interaction was not significant [*F*(1.42, 28.44) = 1.70, *p* = .21. (Fig. 2b).

### Total caloric intake

Mean and Cumulative total caloric intake (chow intake plus sweetened-yogurt intake) was similar between groups in both analysis; *t*(20) = .63, *p* = .54 and *F*(1.38, 27.64) = 1.05, *p* = .34, respectively. This result indicates that chow intake in SAC was perfectly compensatory to the lack of calories in saccharin-sweetened yogurt (Fig. 2c).

### Respiratory variables

#### VO_2_ consumption

**Fig. 3:**
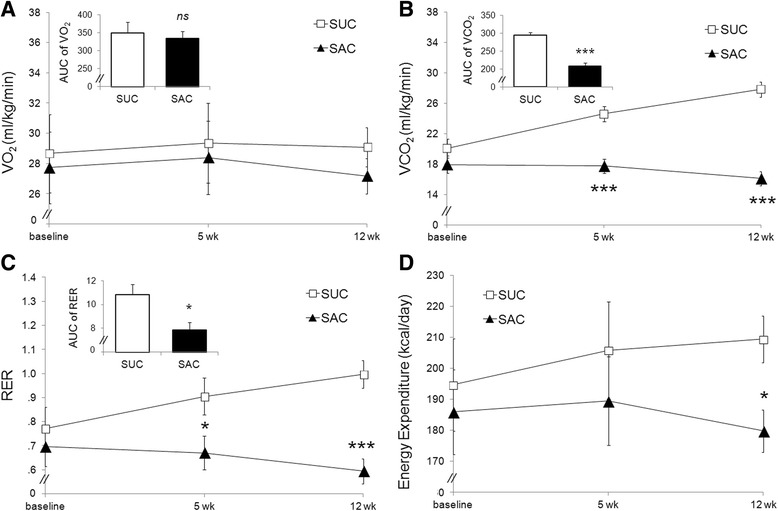
**a**. VO_2_ consumption at baseline, 5th and 12th weeks. Group labels: SAC (Saccharin, *n* = 12) and SUC (Sucrose, *n* = 10). Analyzed by ANOVA of repeated measures [*F*(1, 20.01) = .13, *p* = .72]; bar chart indicate analysis of AUC_(0-12wk)_ by Student *t*-test [*t*(20) = .43, *p* = .67]. Error bars indicate SE. There was no statistical difference. **b**. VCO_2_ production at baseline, 5th and 12th weeks. Group labels: SAC (Saccharin, *n* = 12) and SUC (Sucrose, *n* = 10). Analyzed by ANOVA of repeated measures [*F*(1.42, 28.39) = 14.92, *p* < .001]; bar chart indicate analysis of AUC_(0-12wk)_ by Student *t*-test [*t*(20) = 6.86, *p* = < .001]. Error bars indicate SE. Asterisks indicate *p* ˂ .001. **c**. Respiratory Exchange Ratio (RER) at baseline, 5th and 12th weeks. Group labels: SAC (Saccharin, n = 12) and SUC (Sucrose, *n* = 10). Analyzed by ANOVA of repeated measures [*F*(1.18, 23.59) = 6.26, *p* = .016]; bar chart indicate analysis of AUC_(0-12wk)_ by Student *t*-test [*t*(20) = 2.82, *p* = .011]. Error bars indicate SE. Asterisks indicate: *: *p* ˂ .05; ***: *p* ˂ .001. **d**. Energy expenditure (EE) estimated by Weir's equation at baseline, 5th and 12th weeks. Group labels: SAC (Saccharin, *n* = 12) and SUC (Sucrose, *n* = 10). Analyzed by ANOVA of repeated measures. There was statistical difference between groups on 12th week (*p* = .009). Error bars indicate SE. Asterisk indicate *p* ˂ .05

At baseline, 5th and 12th weeks, the VO_2_ consumption was similar between groups; *F*(1, 20.01) = .13, *p* = .72. Further analysis showed that both groups, not differing from baseline. The analysis of AUC_(0-12wk)_ between groups was also similar; *t*(20) = .43, *p* = .67 (Fig. 3a).

#### VCO_2_ production

At baseline, VCO_2_ production was similar between groups. At weeks 5 and 12, VCO_2_ production was significantly greater in SUC group *F*(1.42, 28.39) = 14.92, *p* < .001. VCO_2_ increased progressively from baseline in SUC at weeks 5 and 12 respectively: 23% (*p* < .001) and 39% (*p* < .001), while there was no difference from baseline in SAC group. The area under the curve (AUC_(0-12wk)_) was 42% greater in SUC group *t*(20) = 6.86, *p* < .001 (Fig. 3b).

### Respiratory Exchange Ratio (RER)

At baseline, RER was similar between groups. At weeks 5 and 12 it was significantly lower in SAC than in SUC (*p* = .036 and *p* < .001, respectively); it was also confirmed when we analyzed AUC_(0-12wk)_; *t*(20) = 2.82, *p* = .011.

When we compared week 12 from baseline there was an increase in 29% on SUC and a decrease in 15% on SAC, but without statistical difference (*p* = .06 and *p* = .67, respectively) (Fig. 3c).

### Energy Expenditure (EE)

At baseline there was no difference in respect of EE between groups: SUC: 194.66 ± 14.95 *vs* SAC: 185.92 ± 13.65 kcal/day (*p* = .67). At week 12 (maximum difference peak), EE was 16% greater in SUC than in SAC group (*p* = .009): SUC: 209.42 ± 7.49 *vs* SAC: 179.82 ± 6.84 kcal/day. In relation to baseline, SUC increased EE in 8% (*p* < .001) while SAC decrease EE in 3% (*p* = .04) (Fig. 3d).

### Correlation between Total Weight Gain and Change in Energy Expenditure (EE)

**Fig. 4:**
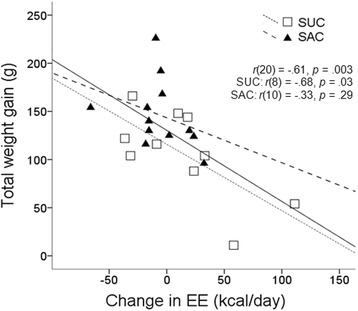
Relationship between total weight gain (g) and change in energy expenditure (kcal/day) observed over the entire experiment demonstrated a significant and inverse correlation [*r*(20) = −.61, *p* = .003] when analyzing both groups. In the stratified analysis we verified a similar correlation in the SUC group [*r*(8) = −.68, *p* = .03] but not in the SAC group [*r*(10) = −.33, *p* = .29]

Considering both groups together, there was a strong and negative correlation [*r*(20) = −.61, *p* = .003] between total weight gain and the change (delta) in energy expenditure from baseline to 12 weeks. However when analyzing the groups separately, we found that SUC maintained a strong and significant inverse correlation [*r*(8) = −.68, *p* = .03], while SAC did not [*r*(10) = −.33, *p* = .29] (Fig. 4).

## Discussion

The present study confirmed our former observations [10, 11] that rats consuming saccharin-sweetened yogurt had greater cumulative weight gain compared to rats eating sucrose-sweetened yogurt, when chow intake is not limited. Nonetheless, this increase was not related to differences in caloric intake, but rather to differences in EE between groups.

We observed that EE increased significantly (*p* < .001) from baseline in rats eating SUC, and decreased slightly (*p* = .04) in rats eating SAC, causing a significant difference between groups at 12 week (*p* = .009). This difference was primarily determined by a significant increase in carbon dioxide production (VCO_2_) in SUC group at weeks 5 and 12 from baseline, combined with a stable oxygen consumption (VO_2_) in both groups. The change in EE (difference from week 12 to week 0), presented as a strong and inverse correlation with the rate of weight gain, which was observed in SUC group (*p* = .03) but not in SAC group (*p* = .29). This suggests that increases in EE could explain the observed differences in weight gain between groups.

As rats were tested in the early post-absorptive period, an increase in EE was expected to be due to the nutrient absorption and digestive metabolism. So, it was interesting that, in contrast to SUC, EE in SAC group did not rise. This effect could be explained to either a lack of carbohydrate intake in SAC group or to a possible direct blunting effect in EE due to saccharin after food intake. As these differences were progressively seen along time, we inferred that they were likely to be a chronic-adaptive mechanism rather than an acute effect simply determined by a single meal deprived of sugar. It is also reasonable to speculate that metabolism adjustments might have occurred in rats using saccharin due to long-term sugar restriction. Future studies, however, are needed to clarify this hypothesis.

We were not able to find similar studies comparing EE between saccharin and sucrose in experimental models in literature. In an interesting study of Swithers *et al*., Sprague–Dawley rats fed for a long time with artificial-sweetened yogurt presented smaller increases in body temperature after an acute single standard meal compared with rats that were fed with glucose-sweetened yogurt during the same period. These authors observed a possible blunting thermal response to food in rats eating saccharin, suggesting that metabolic adjustments of energy expenditure could occur, which is completely in agreement to the data in the present study [9].

Plausible biological mechanisms for explaining EE differences between groups are speculative. During aerobic glucose oxidation, oxygen consumption usually rises proportionally to carbon dioxide production [15] and a parallel rise between VO_2_ and VCO_2_ curves is expected. Nevertheless, as in the present study, VCO_2_ production curve rose more steeply than the VO_2_ curve, suggesting that the anaerobic glycolysis could be the predominant metabolic pathway in SUC rats. This is supported by the fact that RER was near 1 in SUC group and near 0.7 in SAC group, suggesting that carbohydrates were the main fuel used by SUC rats, while fat was predominantly used by SAC rats.

This study has some important strengths. We conducted the experiment in the early post-absorptive state, which is important to minimize the animal stress and the ketogenesis due to the prolonged fasting state, which might interfere in results. It is also important that we measured carbon dioxide directly, and all respiratory variables were measured in duplicate, obtained in the following day, which considerably reduced EE determination variability. Finally, rats were kept in individual cages, which allowed accurate control of caloric intake. One potential limitation in this study was that the Weir formula was originally designed to calculate energy expenditure in humans, although it was later popularized for experimental research [14].

## Conclusion

We concluded that long-term saccharin use may decrease energy expenditure at rest in the post-absorptive, period relative to sucrose in Wistar rats. On the other hand, long-term sucrose intake can increase energy expenditure in rats. This combined effect could explain, at least partially, the weight gain associated to saccharin in relation to sucrose in these animals.

## Abbreviations

AUC: Area under curve
EE: Energy expenditure
FIPE: Fundo de Incentivo à Pesquisa
HCPA: Hospital de Clínicas de Porto Alegre
n.s.: Not significant
RER: Respiratory exchange ratio
SAC: Saccharin group
SE: Standard error
SP: São Paulo
SUC: Sucrose group
UFCSPA: Universidade Federal de Ciências da Saúde de Porto Alegre
UFRGS: Universidade Federal do Rio Grande do Sul
VCO_2_: Volume of carbon dioxide produced
VO_2_: Volume of oxygen consumed
vs: Versus
wk: Week
